# CD47 expression and CD163^+^ macrophages correlated with prognosis of pancreatic neuroendocrine tumor

**DOI:** 10.1186/s12885-021-08045-7

**Published:** 2021-03-25

**Authors:** Rami Imam, Qing Chang, Margaret Black, Caroline Yu, Wenqing Cao

**Affiliations:** Department of Pathology, New York University Grossman School of Medicine, 560 1st Ave, Tisch 4-15I, New York, NY 10016 USA

**Keywords:** Pancreatic tumor, Microenvironment, M2 macrophage, Prognosis, Targeting CD47 therapy

## Abstract

**Background:**

Recent studies have suggested the important roles of CD47 and tumor-associated macrophages in the prognosis and immunotherapy of various human malignancies. However, the clinical significance of CD47 expression and CD163+ TAMs in pancreatic neuroendocrine tumor (PanNET) remains unclear.

**Methods:**

In this study, 47 well-differentiated PanNET resection specimens were collected. CD47 expression and CD163+ macrophages were evaluated using immunohistochemistry and correlated with clinicopathologic properties.

**Results:**

Positive CD47 staining was seen in all PanNETs as well as adjacent normal islets. Compared to normal islets, CD47 overexpressed in PanNETs (*p* = 0.0015). In the cohort, lymph node metastasis (LNM), lymphovascular invasion (LVI), and perineural invasion (PNI) were found in 36.2, 59.6, and 48.9% of the cases, respectively. Interestingly, PanNETs with LNM, LVI, or PNI had significantly lower H-score of CD47 than those without LNM (*p* = 0.035), LVI (*p* = 0.0005), or PNI (*p* = 0.0035). PanNETs in patients with disease progression (recurrence/death) also showed a significantly lower expression of CD47 than those without progression (*p* = 0.022). In contrast, CD163+ macrophage counts were significantly higher in cases with LNM, LVI, and PNI.

**Conclusions:**

Our data suggest relative low CD47 expression and high CD163+ TAMs may act as indicators for poor prognosis of PanNETs.

**Supplementary Information:**

The online version contains supplementary material available at 10.1186/s12885-021-08045-7.

## Background

While the incidence of pancreatic neuroendocrine tumor (PanNET) remains relatively low, accounting for approximately 3% of all pancreatic malignancies, the occurrence of PanNET has risen significantly [1–3]. Over the last four decades, the incidence of PanNET has reportedly increased six-fold in many western nations [1]. Complete surgical resection remains the current mainstay curative therapy for PanNET patients who have localized tumors and limited metastases [4]. Complete remission is rarely achieved in patients harboring lymph node (60–80%) or liver metastasis, or both at the time of presentation [5]. To predict the prognosis, PanNET tumor grade is divided into three categories: low, intermediate and high grade, which are stratified using markers of cellular proliferation, including the mitotic count and/or the Ki-67 proliferation index by immunohistochemistry and morphology based on WHO classification. Five-year survival rates for PanNETs in these three grades were 75, 62, and 7%, respectively [6]. Many patients with low grade PanNETs do not achieve a desired outcome. These facts indicate that new biomarkers are needed to optimize predicting the prognosis of PanNET patients to improve therapeutic strategies for these patients.

The initiation and progression of PanNET are believed to be attributed, at least in part, to the tumor immune microenvironment [7]. Macrophages infiltrating cancer tissues, tumor-associated macrophages (TAMs), are an important component in the tumor microenvironment [8, 9]. Accumulated clinical and experimental evidence have demonstrated the significant roles of TAMs in the growth and progression of tumors [10, 11]. Macrophages can alter its phenotype according to the signal stimulation of the microenvironment. TAMs generally possess characteristics of M2 macrophages such as enhanced expression of CD163 (hemoglobin scavenger receptor) [12, 13]. CD163+ TAMs seem to function as a contributor for tumor growth, metastasis, and poor prognosis. The association of TAMs with a worse prognosis in many malignant tumors has been widely reported [8, 14]. However, except for one recent report that suggested TAMs (CD163+ macrophage) as the most informative prognostic biomarker in PanNET [15], there is still limited information about the role of CD163 positive macrophages in the immune microenvironment in the progression and treatment of PanNET. Furthermore, with the immunotherapy becoming more available and showing promising therapeutic potential in the treatment of malignant tumors, further understanding of the role of CD163+ macrophages (major immune cell type in TAM) in PanNET prognosis will assist physicians to optimize treatment options.

Targeting immunosuppressive checkpoints has been one of the most significant advances in cancer treatment. Agents against PD-L1, CTLA-4, or CD47 have been emerging as potential immunotherapies [16, 17]. CD47 interacts with signal-regulatory proteins (SIRPα) on phagocytes like macrophages to function as a negative immune checkpoint to send a “don’t eat me” signal to ensure that cells are not inappropriately phagocytosed [18]. Data has suggested that CD47 is overexpressed in many human malignancies, such as ovarian cancer, glioma, squamous cell carcinoma, melanoma, osteosarcoma and myeloid leukemia, and its high expression correlates with poor prognosis [19–23]. Since the higher expression of CD47 is believed to bestow cancer cells to evade phagocytic elimination by innate immune cells, targeting CD47 has become more intriguing option for a novel cancer treatment [24–26]. The potential therapeutic efficacies in a variety of cancers are inconsistent, though the data resulted from animal models, pre-clinical and even clinical trials are mostly encouraging [19, 26, 27]. In fact, recent studies have suggested that expression of CD47 in cancer might vary among different cancer types. In fibrolamellar hepatocellular carcinoma [28] and gastric cancer [29]**,** higher CD47 expression has not been seen. Furthermore, increased CD47 mRNA level is not a poorly prognostic factor in gastric tumors [29]. More studies should be performed to elucidate the respective role(s) of CD47 in different cancer types so as to optimize potential anti-CD47 therapy.

There are a few studies on CD47 expression in PanNETs. One report has suggested CD47 overexpression in the CD90^high^ cells of PanNET (cancer stem cell) and is associated with decreased survival of PanNET cells [30]. In the current study, the resection tissue samples from 47 PanNET patients were collected. CD163 and CD47 were stained with specific antibodies. CD163+ macrophage counts and CD47 staining level were correlated with clinicopathologic characteristics to evaluate the significance of CD47 and CD163+ TAMs in PanNET.

## Methods

### Patient recruitment and tissue samples

The pathology database at New York University Langone Health was queried for PanNET diagnosed from major surgical resections that occurred from 2007 to 2017 with the search terms “pancreas”, “neuroendocrine tumor”, “neuroendocrine neoplasm”, “pancreatectomy” and “Whipple procedure”. The selection criteria for cases to be studied included the following: (1) primary neuroendocrine tumor of the pancreas without other coexisting malignancies (2) pathologic stage 1 to 3 tumor with or without lymph node metastasis, (3) documented clinical follow-up of at least 6 months, and (4) availability of slides and tissue blocks for investigation. Cases with familiar syndrome, co-existing other malignancies, small cell carcinoma, large cell neuroendocrine carcinoma, and microadenomas were excluded. A total of 47 well-differentiated PanNET resection specimens were identified. The cases selected were initially diagnosed microscopically by GI pathologists using H&E slides, immunohistochemistry stains (synaptophysin, chromogranin, and ki-67), and staged according to AJCC staging criteria prior to 2017.

The diagnosis of neuroendocrine tumor(s) for each case was confirmed by a GI pathologist (WC), and paraffin blocks were selected (WC and RI) for further study. Clinical data collected on each case included age at the time of diagnosis, gender, ethnicity, follow-up radiology and/or pathology, and survival. With regards to details of the tumor, data collected utilized data from the pathology report such as tumor grade, tumor size, mitotic count, Ki-67 proliferation index, lymph node status, and the status and site of distant metastases. The stage of tumor was adjusted according to AJCC 8th edition. Human tissue specimens in the form of formalin-fixed, paraffin-embedded tissue blocks were collected by the Center for Biospecimen Research and Development (CBRD) under the protocol approved by the New York University School of Medicine Institutional Review Board (NYU SOM IRB).

### Immunohistochemical staining

Immunohistochemical staining was processed by an automatic Ventana Benchmark Immunostainer (Ventana Medical Systems Discovery, Tucson, AZ) at NYU Langone Health Experimental Pathology Research Laboratory [31]. Briefly, the tissues blocks from the selected cases were retrieved. 4-μm sections were deparaffinized, and endogenous peroxidase activity was blocked with 3% hydrogen peroxide for 4 min. Antigen retrieval was performed using Cell Conditioner 2 (Citrate pH 6.0) for 36 min at 95 °C. Unconjugated, rabbit anti-human CD47 (Novus Biologicals Cat# NBP2–32031) and mouse anti-human CD163 (clone MRQ-26, Ventana Medical Systems USA, Cat# 760–4437) were used to detect their expressions. The slides were first incubated for 12 h at room temperature with CD47 antibody (1:100) or for 60 min at 37 °C with CD163 antibody, and secondary anti-rabbit or anti-mouse HRP conjugated antibody was then applied for 8 min. After that, slides were treated with 3,3 diaminobenzidene for 8 min, copper sulfate for 4 min, then were washed in distilled water. All sections were counterstained with hematoxylin and mounted with permanent media. Positive and negative (diluent only) controls were run in parallel with the study sections.

### Evaluation of immunohistochemical staining

CD47 expression level was quantified using the H-score method as described in a previous report [32]. Briefly, weak immunodensity staining received a score of 1, moderate immunoreactivity represented a score of 2, and strong intensity of immunostaining granted a score of 3. The percentage of tumor cells stained at a given staining density was multiplied by the respective numeric score of staining intensity and the components were added to quantify the total H-score. To count CD163 positive macrophages in the stroma of PanNET, the method described in the previous study was adapted [8]. In short, CD163 immunostained slides were first viewed at low magnification (X100), and five areas with high macrophage density in the tumor stroma were selected. CD163+ macrophages in each area were counted at high magnification (X400) and the mean CD163+ macrophage count (MC) was calculated for each case. The composite H-score, or CD163+ MC was evaluated independently by two of three pathologists (WC, RI and QC). Agreement was obtained when the H-scores or CD163+ counts differed by 5% or less. A disparity of more than 5% was re-calculated after consensus review.

### Statistical analysis

Statistical analyses were performed using the Prism 5 statistical package from GraphPad Software, Inc. (La Jolla, CA, USA). The data was expressed as mean ± SE. The correlation between H-score of CD47 staining, or CD163+ cell count and clinicopathologic characteristics was assessed using unpaired t-test and one-way ANOVA. The correlation between CD47 expression and CD163+ cell counts was analyzed with Pearson’s test. A *p*-value less than 0.05 was considered statistically significant.

## Results

### CD47 and CD163 IHC staining in PanNETs

**Fig. 1:**
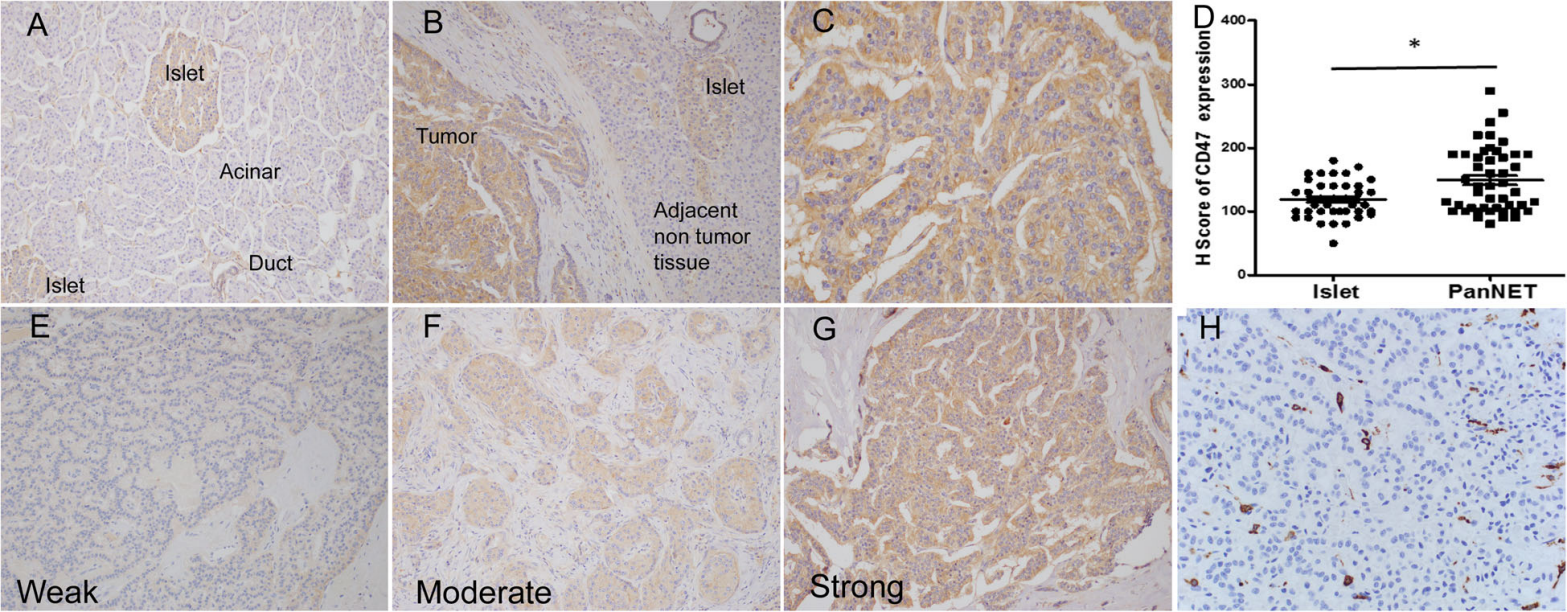
Characteristics of CD47 in human normal pancreas and PanNETs detected by IHC. **a**. CD47 expression in normal pancreatic tissue (X100). **b**. representative image of CD47 expression in PanNET and adjacent normal pancreatic tissue (X200). **c**. CD47 expression on cell membrane and in cytoplasm at high power view (X400). **d**. H-score of CD47 in normal islets and PanNETs. **e**, **f** and **g** showed the intensity of CD47 staining in PanNETs (X200). **h**. CD163+ TAMs in PanNETs (X400). * Significant difference, *p* < 0.05 with unpaired *t* test

Representative images of CD47 staining were displayed in Fig. 1a. In normal pancreas, CD47 was undetectable in acinar and ductal cells. However, weak to moderate CD47 expression was seen in normal pancreatic islet cells. Overexpression of CD47 was observed in all PanNET tumors, but the adjacent non tumor tissue (except islets) showed little to no staining (Fig. 1b). CD47 staining in PanNETs was diffuse in all tumor cells with membranous and cytoplasmic staining (Fig. 1c). The mean H-score of CD47 in PanNETs was significantly higher than that in adjacent islet cells (Fig. 1d, 148.8 ± 7.4 VS 118.6 ± 4.7, *p* = 0.0015). The expression of CD47 varied from weak to strong in PanNETs (Fig. 1e, f and g). CD163+ macrophage staining can be obviously seen in tumor stroma (Fig. 1h).

### Clinicopathologic features and the associations with CD163+ macrophages and CD47 expression

In the cohort, 63.8% (30/47) of patients were males. The median age was 65 years. All tumors were non-functional PanNETs with an average size of 3.2 cm. The percentage of grade 1, 2 or 3 tumors in the cohort was 36.2, 53.2 or 10.6%, respectively. 25.5, 44.7 and 29.8% of tumors corresponded to stage 1, 2 and 3, respectively. In Table 1, the correlations between CD47 expression and CD163+ TAMs with clinicopathologic characteristics were listed. Compared to the group with lower than median age, higher average CD163+ macrophage count and lower CD47 expression (indicated by H-score) was seen in the group with higher than median age.

**Table 1:** Expression of CD163 or CD47 in PanNETs and their associations with clinicopathologic characteristics

	Number of patients	CD163 positive macrophages (mean ± SD)	*P* value	CD47 H- score (mean ± SD)	P value
Age (Years)					
≥ 65	24	82.53 ± 8.54	0.041	134.6 ± 8.89	0.048
< 65	23	59.38 ± 6.81		163.7 ± 11.35	
Gender					
Male	30	70.99 ± 6.64	0.962	159.5 ± 10.21	0.055
Female	17	71.57 ± 10.81		130.0 ± 8.27	
Tumor size (cm)					
≥ mean size (3.2)	20	73.47 ± 10.69	0.735	135.8 ± 12.01	0.130
< ≥mean size	27	69.52 ± 6.15		158.5 ± 9.09	
Ki 67					
≥ 3%	28	71.30 ± 8.01	0.983	140.2 ± 10.00	0.159
< 3%	19	71.05 ± 7.97		161.6 ± 10.53	
Mitosis					
≥ 2/10hpf	28	79.51 ± 8.21	0.076	137.3 ± 9.47	0.059
< 2/10hpf	19	58.96 ± 6.49		165.8 ± 11.05	
Tumor grade					
G1	17	68.24 ± 7.95	0.896	160.6 ± 11.46	0.268
G2	25	67.67 ± 7.27		146.8 ± 10.84	
G3	5	76.70 ± 24.23		119.0 ± 16.61	
Pathologic stage (AJCC 8th)					
1	12	61.12 ± 6.42	0.061	165.4 ± 13.28	0.185
2	21	69.99 ± 9.18		147.4 ± 10.35	
3	14	81.67 ± 12.07		136.8 ± 15.74	

### Lower CD47 expression correlated with LNM, LVI, PNI, or recurrence/death

**Fig. 2:**
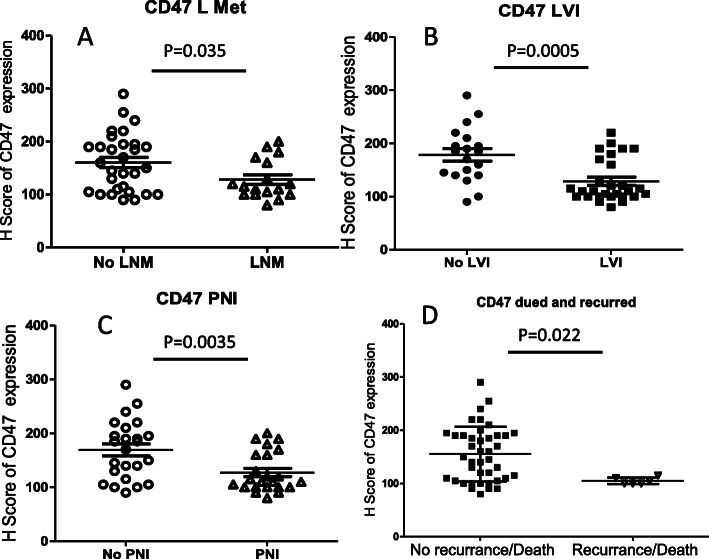
Association of CD47 expression with PanNET prognosis. **a**, **b** and **c** showed the associations of CD47 H-score with LNM, LVI and PNI. **d**. CD47 expression related to recurrence/death

At the time of resection, 36.2, 59.6 and 48.9% of the total cases were found to have LNM, LVI and PNI, respectively. CD47 expression in PanNETs (indicated by H-score) with LNM was significant lower than that without LNM (128.2 ± 9.0 VS 160.5 ± 9.9, *p* = 0.035; Fig. 2a). In PanNETs with LVI or PNI, the expression of CD47 was significantly decreased compared to the group without corresponding invasion (Fig. 2b-c). There was no obvious difference in CD47 expression among patients with LNM, LVI and PNI. On follow-up, 4 patients developed recurrent diseases and 2 patients died of PanNET. Significantly lower CD47 expression was noticed in PanNETs in patients with recurrent disease/death compared to those without recurrent disease/death (105.0 ± 2.6 VS 155.2 ± 8.0, *p* = 0.022, Fig. 2d).

### Higher CD163+ macrophage counts were seen in PanNETs with LNM, LVI, PNI, or recurrence/death

**Fig. 3:**
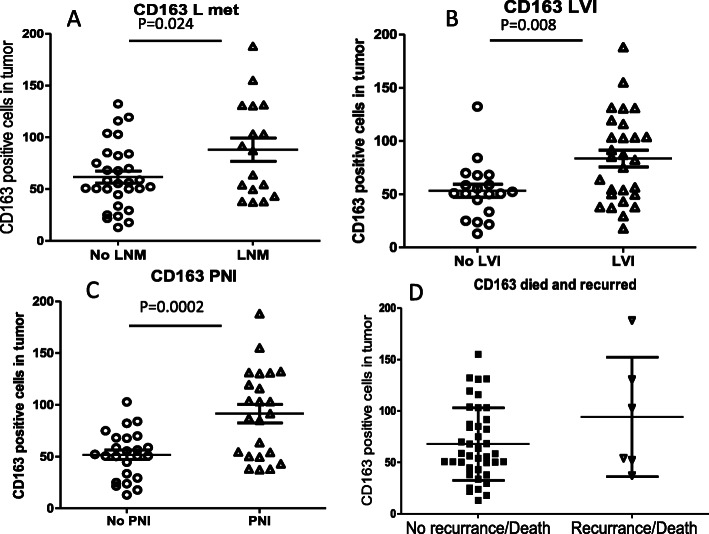
CD163+ TAMs associated with PanNET prognosis. **a**, **b**, and **c** displayed the associations of CD163+ macrophages with LNM, LVI and PNI. **d**. association of CD163+ macrophage counts with recurrence/death

The average count of CD163+ TAMs in PanNETs with LNM, LVI or PNI was significantly higher than that without related metastasis (Fig. 3a-c). Similar CD163+ macrophage counts were seen in group with LNM, LVI and PNI. Although there was a trend of increasing CD163+ macrophage counts in PanNETs in patients with recurrences/deaths compared to those without recurrence/death (94.1 ± 23.7 VS 67.8 ± 5.5, Fig. 3d), the data was not statistically significant.

Notably, studies have shown CD47 expression might be associated with macrophage infiltration, and CD47 reduction could increase macrophage infiltration in pancreas. One recent study on non-small cell lung cancer suggested that CD47 blockade could enhance macrophage infiltration [33]. Herein, we did regression assay between CD163+ cell counts and CD47 expression using Pearson’s test. The results from the assay showed a negative correlation between CD163+ macrophage count and CD47 expression (r = − 0.34). A high CD163+ macrophage count associated with low expression of CD47 (*p* = 0.031, supplemental Figure 1). However the data needed further studies with larger cohort to confirm.

## Discussion

In this study, we first reported CD47 expression in human normal pancreatic tissue. We found weak to moderate CD47 staining in the normal pancreatic islet cells, but not in acinar or ductal cells. This was similar to the reported CD47 expression in normal mice pancreas [34]. In PanNETs, CD47 was overexpressed; its staining in tumor tissue was not only obviously higher than that in adjacent non-tumor tissue, but also significantly higher than that in islet cells. Relative low expression of CD47 in PanNETs was associated with tumor metastasis trends. CD163+ macrophages were seen in the stroma of tumor with variable numbers, and a higher CD163+ macrophage count was related to LNM, LVI and PNI.

Overexpression of CD47 in tumor cells compared to non-malignant cells has been demonstrated in multiple studies with various types of malignancies, which was believed to warrant cancer cells to exploit the “don’t eat me” function of CD47 [26]. Krampitz et al. showed that CD47 was highly expressed in cells from human PanNET compared to surrounding noncancerous pancreatic cells via flow cytometry [30]. In line with their findings, in this study, the expression of CD47 in PanNETs detected by IHC was conspicuously higher than that in adjacent non-tumor tissues. Considering the potential origin of PanNET [35], we further compared CD47 expression in normal pancreatic islets with that in PanNETs. CD47 expression displayed a significantly increased expression in PanNETs, providing more evidence for the concept that the increase of CD47 expression in tumor would aid escape immunosurveillance of the host. Notably, recent studies have indicated that CD47 expression appeared to vary among different types of cancers. For example, in fibrolamellar subtype of hepatocellular carcinoma, CD47 expression did not increase compared to normal hepatic tissue [28]. Moreover, in gastric carcinoma, no overexpression of CD47 was found when compared to adjacent non-tumor gastric tissue [29].

High expression of CD47 would help cancer cells evade macrophage-mediated phagocytosis and is associated with growth and progression of many types of cancers. Data from several types of cancers have illustrated the positive correlation between high expression of CD47 in tumor cells and poor prognosis [36–38]. However, it remains controversial whether high CD47 expression could serve as an indicator for poor outcome of patients. In gastric cancer, in addition to non-overexpression compared to normal gastric tissue, the high mRNA of CD47 was also not associated with poor prognosis [29]. In non-small cell lung cancer, CD47 overexpression was not a prognostic factor [39]. Data in this study with PanNETs indicated a relative lower level of CD47 expression was not only associated with LNM, PNI and LVI, but also associated with PanNET recurrence and patient death. These evidence suggested that the association of CD47 expression with patient’s prognosis might be dependent on a specific type of cancer. In PanNET, relative lower expression of CD47 may indicate a poor prognosis.

It is worth to mention that CD47 may mediate many different biologic activities besides its immunoregulatory functions. CD47 is not only a receptor for SIRP and the matricellular protein thrombospondin-1 (TSP-1), but also for ligands that are emerging [40]. Evidence has indicated that CD47 is a versatile factor in cell activity, such as in angiogenesis [41, 42], cell migration, immune-cell filtration [43], as well as cell death [44]. In CD47 deficient mice, angiogenesis in xenograft tumor was enhanced, and the lack of CD47 accelerated tumor progression [45]. These findings suggested that further studies on pathologic and therapeutic roles of CD47 and its related signaling pathways in PanNETs may be needed.

Evidence has indicated that the change of macrophage subtypes in tumor microenvironment has important roles in tumor progression and metastasis [11, 46]. Heterogeneity of macrophage phenotypes is observed among TAMs in various malignant tumors, but CD163+ TAMs may be the main population [8, 47, 48]. A higher CD163+ macrophage count or an increase in M2/M1 Ratio in a variety of cancers indicates a poor prognosis or metastasis [8, 48], although it remains unclear how CD163 works in the protumoral activation of TAMs. In PanNETs, our report revealed that a higher CD163+ cell count related to LNM, PNI and LVI, suggesting CD163+ TAM as an indicator for poor prognosis. The data supports the previous report showing the association of higher CD163+ macrophage with a worse disease free survival and disease specific survival in PanNETs [15]. It is worth to mention that in this study that CD163 + macrophages in the group with recurrence and/or death was not significantly higher, compared to that without recurrence and/or death. The difference with the previous report [15] may result from our small sample size.

Escaping immunosurveillance of the host is an essential step for tumor development and metastasis. Overexpression of immunosuppressive molecules may act as a key mechanism under this immune absconsion [49–51]. CD47, an inhibitory receptor expressed on cell surface, mediates signaling to aid malignant cells in avoiding phagocytosis via interacting with SIPRα of immune cells such as macrophages. Accumulating data from laboratory and clinical studies have not only revealed highly expressed CD47 in many types of cancers, but also provided encouraging data for the novel therapeutic strategy by targeting CD47 [26, 52, 53]. However, clinical trials have given rise to some uncertainty, even controversial data on the efficacy of the potential immune therapy [19], suggesting that further understanding related mechanisms underlying CD47 functions, relationship with immune cells and responses in tumors may be required. Regarding expression of CD47 in tumors, overexpression of CD47 was not exhibited in all kinds of cancers, and non-overexpression has been found in gastric cancer [29] and fibrolamellar subtype of hepatocellular carcinoma [28], implying that targeting CD47 therapy might not be suitable for these types of malignancies. In one report, using engraft animal models established via injecting the selected CD90^high^ PanNET cell (cancer stem cell) with higher expression of CD47, anti-CD47 antibody inhibited PanNET tumor growth in animal model [30]. However, our data indicated that CD47 expression was relative lower in PanNETs with poor prognosis, suggesting that targeting CD47 therapy might not be ideal treatment for the patients with metastasis and poor prognosis.

Based on our knowledge, there seems to be no study to clearly show the relationship of CD47 expression on cancer cells with stromal CD163+ macrophages. In the study, both higher CD163+ macrophages and low expression of CD47 in PanNETs were associated with metastasis status. A recent study on diabetes showed that decreased CD47 expression enhanced the immune cell infiltration into islets [26]. Further investigation on the relationship between CD47 expression and CD163+ macrophages in tumor might assist to justify the efficacy of targeting CD47 therapeutic approach in PanNETs.

This study has some limitations. It was a retrospective study using resection tissues from a small cohort in one medical center. In this study, only morphologic analysis and IHCs were adopted for experiments. More studies should be performed to confirm or extend the results. We could expect several translational applications of our results in the future studies. First, the expression of CD47, CD163+ macrophages, and their prognostic value should be verified in a large independent clinical cohort, ideally in a prospective study. Second, according to our results, while exploring CD47 targeting therapy in PanNET, in additions to its effect on phagocytosis, other effects of CD47 on angiogenesis, cell death and migration may need to be tested. Moreover, future effort may be needed to illustrate the potential prognostic role of correlation between CD47 expression and CD163+ macrophage counts.

## Conclusions

In summary, the data from the study revealed that CD47 was overexpressed in PanNETs. A relatively lower expression of CD47 in tumor cells or higher CD163+ TAMs was related to poor prognosis, suggesting that they might act as indicators for PanNET prognosis. Suppressing CD47 might be inappropriate therapy for PanNET with metastasis.

## Supplementary Information

**Additional file 1: Figure S1.** Correlation between CD47 expression and CD163+ macrophage counts.

## Data Availability

The datasets used and/or analysed during the current study available from the correspondingauthor on reasonable request.

## Abbreviations

PanNET: Pancreatic neuroendocrine tumor
TAM: Tumor associated macrophage
LNM: Lymph node metastasis
LVI: Lymphovascular invasion
PNI: Perineural invasion
IHC: Immunohistochemistry

## References

[1] Dasari A, Shen C, Halperin D, Zhao B, Zhou S, Xu Y, Shih T, Yao JC (2017). Trends in the incidence, prevalence, and survival outcomes in patients with neuroendocrine tumors in the United States. JAMA Oncol.

[2] Franko J, Feng W, Yip L, Genovese E, Moser AJ (2010). Non-functional neuroendocrine carcinoma of the pancreas: incidence, tumor biology, and outcomes in 2,158 patients. J Gastrointest Surg.

[3] Rindi G, Falconi M, Klersy C, Albarello L, Boninsegna L, Buchler MW, Capella C, Caplin M, Couvelard A, Doglioni C, Delle Fave G, Fischer L, Fusai G, de Herder WW, Jann H, Komminoth P, de Krijger RR, la Rosa S, Luong TV, Pape U, Perren A, Ruszniewski P, Scarpa A, Schmitt A, Solcia E, Wiedenmann B (2012). TNM staging of neoplasms of the endocrine pancreas: results from a large international cohort study. J Natl Cancer Inst.

[4] Krampitz GW, Norton JA (2013). Pancreatic neuroendocrine tumors. Curr Probl Surg.

[5] Modlin IM, Oberg K, Chung DC, Jensen RT, de Herder WW, Thakker RV, Caplin M, Delle Fave G, Kaltsas GA, Krenning EP, Moss SF, Nilsson O, Rindi G, Salazar R, Ruszniewski P, Sundin A (2008). Gastroenteropancreatic neuroendocrine tumours. Lancet Oncol.

[6] Cives M, Strosberg J (2014). An update on gastroenteropancreatic neuroendocrine tumors. Oncology (Williston Park).

[7] Cives M, Pelle E, Quaresmini D, Rizzo FM, Tucci M, Silvestris F (2019). The tumor microenvironment in neuroendocrine tumors: biology and therapeutic implications. Neuroendocrinology.

[8] Cao W, Peters JH, Nieman D, Sharma M, Watson T, Yu J (2015). Macrophage subtype predicts lymph node metastasis in oesophageal adenocarcinoma and promotes cancer cell invasion in vitro. Br J Cancer.

[9] Shiraishi D, Fujiwara Y, Horlad H, Saito Y, Iriki T, Tsuboki J, Cheng P, Nakagata N, Mizuta H, Bekki H, Nakashima Y, Oda Y, Takeya M, Komohara Y (2018). CD163 is required for Protumoral activation of macrophages in human and murine sarcoma. Cancer Res.

[10] Mantovani A (2018). Redundancy and robustness versus division of labour and specialization in innate immunity. Semin Immunol.

[11] Qian BZ, Pollard JW (2010). Macrophage diversity enhances tumor progression and metastasis. Cell.

[12] Mantovani A, Marchesi F, Malesci A, Laghi L, Allavena P (2017). Tumour-associated macrophages as treatment targets in oncology. Nat Rev Clin Oncol.

[13] Mantovani A, Sozzani S, Locati M, Allavena P, Sica A (2002). Macrophage polarization: tumor-associated macrophages as a paradigm for polarized M2 mononuclear phagocytes. Trends Immunol.

[14] Jeong H, Hwang I, Kang SH, Shin HC, Kwon SY (2019). Tumor-associated macrophages as potential prognostic biomarkers of invasive breast Cancer. J Breast Cancer.

[15] Cai L, Michelakos T, Deshpande V, Arora KS, Yamada T, Ting DT, Taylor MS, Castillo CF, Warshaw AL, Lillemoe KD, Ferrone S, Ferrone CR (2019). Role of tumor-associated macrophages in the clinical course of pancreatic neuroendocrine tumors (PanNETs). Clin Cancer Res.

[16] Sharpe AH, Pauken KE (2018). The diverse functions of the PD1 inhibitory pathway. Nat Rev Immunol.

[17] Veillette A, Chen J (2018). SIRPalpha-CD47 immune checkpoint blockade in anticancer therapy. Trends Immunol.

[18] Liu L, Zhang L, Yang L, Li H, Li R, Yu J, Yang L, Wei F, Yan C, Sun Q, Zhao H, Yang F, Jin H, Wang J, Wang SE, Ren X (2017). Anti-CD47 antibody as a targeted therapeutic agent for human lung Cancer and Cancer stem cells. Front Immunol.

[19] Huang Y, Ma Y, Gao P, Yao Z (2017). Targeting CD47: the achievements and concerns of current studies on cancer immunotherapy. J Thorac Dis.

[20] Lin GHY, Chai V, Lee V, Dodge K, Truong T, Wong M, Johnson LD, Linderoth E, Pang X, Winston J, Petrova PS, Uger RA, Viller NN (2017). TTI-621 (SIRPalphaFc), a CD47-blocking cancer immunotherapeutic, triggers phagocytosis of lymphoma cells by multiple polarized macrophage subsets. PLoS One.

[21] Majeti R, Chao MP, Alizadeh AA, Pang WW, Jaiswal S, Gibbs KD, van Rooijen N, Weissman IL (2009). CD47 is an adverse prognostic factor and therapeutic antibody target on human acute myeloid leukemia stem cells. Cell.

[22] Yang K, Xu J, Liu Q, Li J, Xi Y (2019). Expression and significance of CD47, PD1 and PDL1 in T-cell acute lymphoblastic lymphoma/leukemia. Pathol Res Pract.

[23] Yoshida K, Tsujimoto H, Matsumura K, Kinoshita M, Takahata R, Matsumoto Y, Hiraki S, Ono S, Seki S, Yamamoto J, Hase K (2015). CD47 is an adverse prognostic factor and a therapeutic target in gastric cancer. Cancer Med.

[24] Tseng D, Volkmer JP, Willingham SB, Contreras-Trujillo H, Fathman JW, Fernhoff NB, Seita J, Inlay MA, Weiskopf K, Miyanishi M, Weissman IL (2013). Anti-CD47 antibody-mediated phagocytosis of cancer by macrophages primes an effective antitumor T-cell response. Proc Natl Acad Sci U S A.

[25] Weissman S, Sebrow J, Gonzalez HH, Weingarten MJ, Rosenblatt S, Mehta TI, Thaker R, Krzyzak M, Saleem S (2019). Diagnosis of primary colorectal carcinoma with primary breast Cancer: associations or connections?. Cureus.

[26] Zhang W, Huang Q, Xiao W, Zhao Y, Pi J, Xu H, Zhao H, Xu J, Evans CE, Jin H (2020). Advances in anti-tumor treatments targeting the CD47/SIRPalpha Axis. Front Immunol.

[27] Michaels AD, Newhook TE, Adair SJ, Morioka S, Goudreau BJ, Nagdas S, Mullen MG, Persily JB, Bullock TNJ, Slingluff CL, Ravichandran KS, Parsons JT, Bauer TW (2018). CD47 blockade as an adjuvant immunotherapy for Resectable pancreatic Cancer. Clin Cancer Res.

[28] Cooney T, Wei MC, Rangaswami A, Xu L, Sage J, Hazard FK (2017). CD47 is not over-expressed in Fibrolamellar hepatocellular carcinoma. Ann Clin Lab Sci.

[29] Sudo T, Takahashi Y, Sawada G, Uchi R, Mimori K, Akagi Y (2017). Significance of CD47 expression in gastric cancer. Oncol Lett.

[30] Krampitz GW, George BM, Willingham SB, Volkmer JP, Weiskopf K, Jahchan N, Newman AM, Sahoo D, Zemek AJ, Yanovsky RL, Nguyen JK, Schnorr PJ, Mazur PK, Sage J, Longacre TA, Visser BC, Poultsides GA, Norton JA, Weissman IL (2016). Identification of tumorigenic cells and therapeutic targets in pancreatic neuroendocrine tumors. Proc Natl Acad Sci U S A.

[31] Vougiouklakis T, Belovarac BJ, Lytle A, Chiriboga L, Ozerdem U (2020). The diagnostic utility of EZH2 H-score and Ki-67 index in non-invasive breast apocrine lesions. Pathol Res Pract.

[32] Cao W, Sharma M, Imam R, Yu J (2019). Study on diagnostic values of astrocyte elevated gene 1 (AEG-1) and Glypican 3 (GPC-3) in hepatocellular carcinoma. Am J Clin Pathol.

[33] Zhang X, Wang Y, Fan J, Chen W, Luan J, Mei X, Wang S, Li Y, Ye L, Li S, Tian W, Yin K, Ju D (2019). Blocking CD47 efficiently potentiated therapeutic effects of anti-angiogenic therapy in non-small cell lung cancer. J Immunother Cancer.

[34] Kobayashi M, Ohnishi H, Okazawa H, Murata Y, Hayashi Y, Kobayashi H, Kitamura T, Matozaki T (2008). Expression of Src homology 2 domain-containing protein tyrosine phosphatase substrate-1 in pancreatic beta-cells and its role in promotion of insulin secretion and protection against diabetes. Endocrinology.

[35] Bocchini M, Nicolini F, Severi S, Bongiovanni A, Ibrahim T, Simonetti G, Grassi I, Mazza M (2020). Biomarkers for pancreatic neuroendocrine neoplasms (PanNENs) management-an updated review. Front Oncol.

[36] Barrera L, Montes-Servin E, Hernandez-Martinez JM, Garcia-Vicente MLA, Montes-Servin E, Herrera-Martinez M, Crispin JC, Borbolla-Escoboza JR, Arrieta O (2017). CD47 overexpression is associated with decreased neutrophil apoptosis/phagocytosis and poor prognosis in non-small-cell lung cancer patients. Br J Cancer.

[37] Chao MP, Alizadeh AA, Tang C, Myklebust JH, Varghese B, Gill S, Jan M, Cha AC, Chan CK, Tan BT, Park CY, Zhao F, Kohrt HE, Malumbres R, Briones J, Gascoyne RD, Lossos IS, Levy R, Weissman IL, Majeti R (2010). Anti-CD47 antibody synergizes with rituximab to promote phagocytosis and eradicate non-Hodgkin lymphoma. Cell.

[38] Rivera A, Fu X, Tao L, Zhang X (2015). Expression of mouse CD47 on human cancer cells profoundly increases tumor metastasis in murine models. BMC Cancer.

[39] Arrieta O, Aviles-Salas A, Orozco-Morales M, Hernandez-Pedro N, Cardona AF, Cabrera-Miranda L, Barrios-Bernal P, Soca-Chafre G, Cruz-Rico G, Pena-Torres ML (2020). Association between CD47 expression, clinical characteristics and prognosis in patients with advanced non-small cell lung cancer. Cancer Med.

[40] Eladl E, Tremblay-LeMay R, Rastgoo N, Musani R, Chen W, Liu A, Chang H (2020). Role of CD47 in hematological malignancies. J Hematol Oncol.

[41] Gao Q, Chen K, Gao L, Zheng Y, Yang YG (2016). Thrombospondin-1 signaling through CD47 inhibits cell cycle progression and induces senescence in endothelial cells. Cell Death Dis.

[42] Kaur S, Roberts DD (2011). CD47 applies the brakes to angiogenesis via vascular endothelial growth factor receptor-2. Cell Cycle.

[43] Sick E, Jeanne A, Schneider C, Dedieu S, Takeda K, Martiny L (2012). CD47 update: a multifaceted actor in the tumour microenvironment of potential therapeutic interest. Br J Pharmacol.

[44] Leclair P, Lim CJ (2020). CD47 (cluster of differentiation 47): an anti-phagocytic receptor with a multitude of signaling functions. Anim Cells Syst (Seoul).

[45] Gao L, Chen K, Gao Q, Wang X, Sun J, Yang YG (2017). CD47 deficiency in tumor stroma promotes tumor progression by enhancing angiogenesis. Oncotarget.

[46] Horimoto Y, Polanska UM, Takahashi Y, Orimo A (2012). Emerging roles of the tumor-associated stroma in promoting tumor metastasis. Cell Adhes Migr.

[47] Heusinkveld M, de Vos van Steenwijk PJ, Goedemans R, Ramwadhdoebe TH, Gorter A, Welters MJ, van Hall T, van der Burg SH (2011). M2 macrophages induced by prostaglandin E2 and IL-6 from cervical carcinoma are switched to activated M1 macrophages by CD4+ Th1 cells. J Immunol.

[48] Komohara Y, Jinushi M, Takeya M (2014). Clinical significance of macrophage heterogeneity in human malignant tumors. Cancer Sci.

[49] Pitt JM, Marabelle A, Eggermont A, Soria JC, Kroemer G, Zitvogel L (2016). Targeting the tumor microenvironment: removing obstruction to anticancer immune responses and immunotherapy. Ann Oncol.

[50] Ribatti D (2017). The concept of immune surveillance against tumors. The first theories. Oncotarget.

[51] Sharma P, Allison JP (2015). Immune checkpoint targeting in cancer therapy: toward combination strategies with curative potential. Cell.

[52] Lian S, Xie X, Lu Y, Jia L (2019). Checkpoint CD47 function on tumor metastasis and immune therapy. Onco Targets Ther.

[53] Matlung HL, Szilagyi K, Barclay NA, van den Berg TK (2017). The CD47-SIRPalpha signaling axis as an innate immune checkpoint in cancer. Immunol Rev.

