# Supplementary material for: CD47 expression and CD163+ macrophages correlated with prognosis of pancreatic neuroendocrine tumor
Source: BMC Cancer. 2021 Mar 25;21:320. doi: 10.1186/s12885-021-08045-7 (PMC7992939; doi:10.1186/s12885-021-08045-7)
Supplement: Supplementary file 1 — Additional file 1: Figure S1. Correlation between CD47 expression and CD163+ macrophage counts. [file 12885_2021_8045_MOESM1_ESM.pptx]

## Slide 1
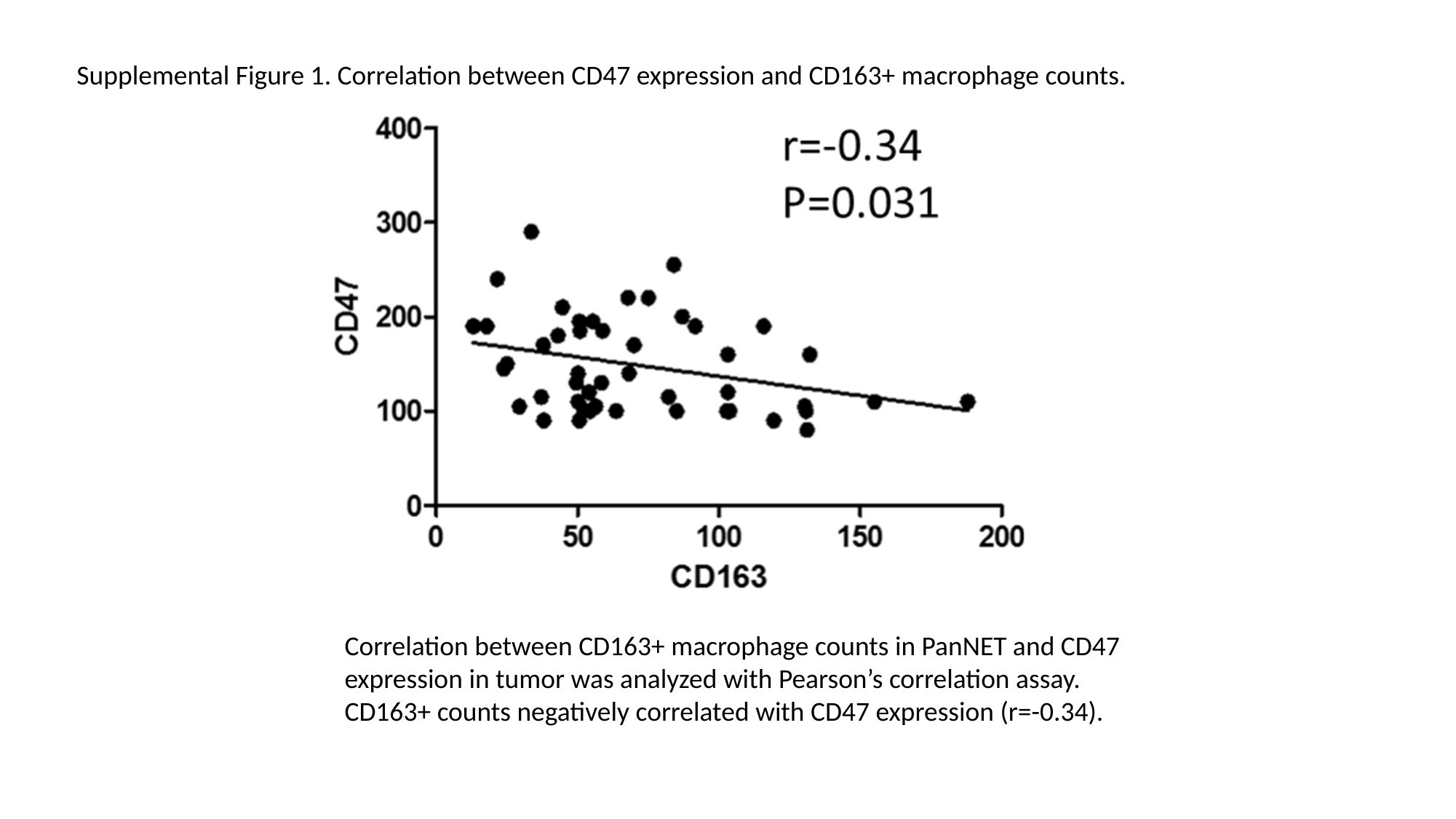

Supplemental Figure 1. Correlation between CD47 expression and CD163+ macrophage counts.
Correlation between CD163+ macrophage counts in PanNET and CD47 expression in tumor was analyzed with Pearson’s correlation assay. CD163+ counts negatively correlated with CD47 expression (r=-0.34).
